# Risk Assessment of Psychotropic Drugs on Mitochondrial Function Using In Vitro Assays

**DOI:** 10.3390/biomedicines11123272

**Published:** 2023-12-11

**Authors:** Alicia Rosell-Hidalgo, Julie Eakins, Paul Walker, Anthony L. Moore, Taravat Ghafourian

**Affiliations:** 1 Cyprotex Discovery Ltd., No. 24 Mereside, Alderley Park, Macclesfield, Cheshire SK10 4TG, UK; a.rosell-hidalgo@cyprotex.com (A.R.-H.); j.eakins@cyprotex.com (J.E.); 2 Department of Biochemistry and Biomedicine, School of Life Sciences, University of Sussex, Falmer, Brighton BN1 9QG, UK; a.l.moore@sussex.ac.uk; 3 Department of Pharmaceutical Sciences, Barry & Judy Silverman College of Pharmacy, Nova Southeastern University, 3200 South University Drive, Ft. Lauderdale, FL 33328-2018, USA

**Keywords:** mitochondrial toxicity, antipsychotics, anticonvulsants, antidepressants, anxiolytic drugs, seahorse

## Abstract

Mitochondria are potential targets responsible for some drug- and xenobiotic-induced organ toxicities. However, molecular mechanisms of drug-induced mitochondrial toxicities are mostly unknown. Here, multiple in vitro assays were used to investigate the effects of 22 psychotropic drugs on mitochondrial function. The acute extracellular flux assay identified inhibitors of the electron transport chain (ETC), i.e., aripiprazole, phenytoin, and fluoxetine, an uncoupler (reserpine), substrate inhibitors (quetiapine, carbamazepine, buspirone, and tianeptine), and cytotoxic compounds (chlorpromazine and valproic acid) in HepG2 cells. Using permeabilized HepG2 cells revealed minimum effective concentrations of 66.3, 6730, 44.5, and 72.1 µM for the inhibition of complex-I-linked respiration for quetiapine, valproic acid, buspirone, and fluoxetine, respectively. Assessing complex-II-linked respiration in isolated rat liver mitochondria revealed haloperidol is an ETC inhibitor, chlorpromazine is an uncoupler in basal respiration and an ETC inhibitor under uncoupled respiration (IC_50_ = 135 µM), while olanzapine causes a mild dissipation of the membrane potential at 50 µM. This research elucidates some mechanisms of drug toxicity and provides some insight into their safety profile for clinical drug decisions.

## 1. Introduction

Adverse drug reactions of several pharmacological drugs have been attributed to their effects on mitochondria. Drugs from a diverse range of classes have been shown to cause drug-induced mitochondrial dysfunction leading to various organ toxicities and have either been withdrawn from the market or received Black Box warnings from the Food and Drug Administration (FDA) [1]. For example, it has been shown that drugs with the potential to cause Drug-Induced Liver Injury (DILI) are likely to be those that inhibit various aspects of mitochondrial function [2]. Despite this, the extent of drug-mitochondrial interactions and the exact mechanisms of such interactions are largely unknown for some pharmacological drugs.

Pharmacological agents can cause mitochondrial toxicity through direct alterations that involve binding to specific mitochondrial targets, for example, the inhibition or uncoupling of the electron transport chain (ETC), inhibition of the transport or oxidation of substrates, inhibition of mtDNA replication, transcription, and translation, or inhibition of the import of mitochondrial proteins encoded by the nucleus [3].

Mitochondrial toxicology has become an area of great interest to the pharmaceutical industry and, in response, high-throughput assays for testing of drug-induced mitochondrial dysfunction have been developed to test compounds in a fast and relatively inexpensive way. Oxygen consumption measurement is an informative experimental technique that can characterize cellular metabolism and mitochondrial function, while also assisting in the identification of the mechanisms of toxicity [4]. Traditionally, an assessment of mitochondrial function was performed using the Clark-type electrode to monitor oxygen consumption, which does not have sufficient throughput for an industrial setting. The Seahorse metabolic flux analyzer, which uses micro-plate-based respirometry, allows efficient testing of drug effects by simultaneously monitoring oxygen consumption, as a measure of mitochondrial respiration, and extracellular acidification rate, as an indirect measure of glycolysis. The Seahorse metabolic flux analyzer can produce robust and reliable data regarding drug effects on mitochondrial function, reducing the amount of sample material required for a single replicate by orders of magnitude relative to platinum-based Clark-type electrodes [5,6].

Several model systems have been used for respirometry studies for mitochondrial function, each with its own strengths and limitations: animals, whole cells, isolated mitochondria, tissue sections, and sub-mitochondrial particles. With isolated mitochondria, some limitations that ought to be considered are the limited time they remain functional post-isolation [7], compromised morphology and structural integrity following manual homogenization of tissue, the disintegration of mitochondrial–cytoskeletal networks and branching structure [8], and the lack of cellular context [9]. However, measuring the respiration in isolated mitochondria is appropriate when examining drug candidates for a mitochondrial mechanism of action to pinpoint precise sites of action [6]. On the other hand, the advantages of using intact cells include an undisturbed cellular environment, greater physiological relevance, and no artifacts due to mitochondrial isolation. Additionally, whole cells allow tracking changes in mitochondrial localization, dynamics, and number. The limitations of intact cells include lack of the in vivo context, poor permeation of many compounds through the cell membrane, and variability due to the choice of reagents, hormones, growth factors, and other substrates depending on the experimental design [9]. As an alternative to isolated mitochondria, various cell permeabilization methods have been developed to allow sufficient permeation of the drug and various mitochondrial respiration substrates. These methods are high throughput, while reducing the amount of biological material required compared with traditional isolated mitochondria assays [10]. However, it seems that there is no single method that can identify all the drugs with mitochondrial liabilities.

The focus of this investigation was the elucidation of the effects of several psychotropic drugs on mitochondria. Psychotropic drugs are used for the treatment of various mental health disorders and include pharmacological classes of antidepressants, anxiolytics, antipsychotics, psychostimulants, and anticonvulsants, among others. These drugs have shown a number of toxicities and side effects that may be caused due to their interaction with mitochondria. The first pharmacological group investigated here consisted of antipsychotics (APs), also known as neuroleptics. These drugs are classified as typical or “first-generation” and atypical or “second-generation” antipsychotics. Acting mainly on dopamine type 2 (D2) receptors, typical APs are commonly prescribed for schizophrenia [11]. However, owing to their high and non-specific occupation of D2 receptors, they are associated with extrapyramidal symptoms and hyperprolactinemia [12]. Second-generation APs have a lower risk of extrapyramidal side effects, with a lower affinity for the D2 receptors and a higher affinity for the serotoninergic and other receptors [13]. However, there is still an ongoing debate as to whether second-generation APs are superior to first-generation APs in terms of both efficacy and toxicity [14,15,16]. The safety advantages of the atypical APs have been questioned as they have been linked to a wide range of side effects, including reproductive dysfunction and metabolic effects, such as weight gain [17,18], dyslipidemia, and diabetes mellitus [19,20,21,22,23]. Clozapine and olanzapine are associated with a higher risk of metabolic side effects [24], followed by the medium-risk drugs quetiapine and risperidone and the low-risk drugs ziprasidone and aripiprazole [25,26,27]. Furthermore, although less frequently than first-generation APs, atypical APs can also induce extrapyramidal side effects [28,29,30]. Although the exact underlying mechanisms of various adverse effects are not well understood, an increasing body of evidence has linked both typical and atypical APs to disturbed mitochondrial function [31,32].

The second group of psychotropic drugs investigated here included several anticonvulsant agents. Anticonvulsants (ACs), also commonly known as antiepileptic or antiseizure drugs, are a diverse group of pharmacological agents that help prevent or treat seizures [33,34,35]. ACs can be broken down into two categories: narrow-spectrum, which are designed for specific types of seizures, and broad-spectrum, which treat a wide variety of seizure types [36]. ACs can have a variety of mechanisms of action. For instance, some inhibit the activation of sodium channels (phenytoin, carbamazepine), while others block calcium channels (valproic acid) or bind to gamma-aminobutyric acid (GABA-A) receptors (phenobarbital) [36,37]. ACs can show various side effects, some of which have been attributed to mitochondrial toxicity [38]. In fact, ACs have been reported to interfere with various mitochondrial pathways, structures, and functions, including the respiratory chain, oxidative phosphorylation (OXPHOS), the tricarboxylic acid (TCA) cycle, and β-oxidation [39]. Valproic acid is the most widely investigated AC in relation to mitochondrial toxicity, probably due to its pronounced liver toxicity and because it causes the most severe side effects in patients with mitochondrial disorders [39,40]. However, other ACs have also been associated with hepatic mitochondrial dysfunction and elucidation of mechanisms of mitochondrial action will require further investigations [41,42].

Other psychotropic drugs including three antidepressants and two anxiolytic drugs were also investigated here. The mechanism of action of antidepressants is very diverse, but selective serotonin reuptake inhibitors (SSRIs) are currently the first-choice drugs for depression therapy [43,44]. Although SSRIs are generally well tolerated, cases of liver injury and other side effects such as nausea, vomiting and sexual disorders have been documented [44,45]. Several SSRIs, including the widely prescribed fluoxetine or sertraline, have been reported to have mitochondrial off-targets and impair mitochondrial function [45].

In summary, although the mechanisms of the pharmacological action of many psychotropic drugs may be well known, their off-target effects on mitochondria and cell energy metabolism remain to be fully elucidated. Hence, in this study, different in vitro assays were performed to investigate the effects of 22 psychotropic drugs, including a series of antipsychotics, anticonvulsants, antidepressants, and anxiolytic drugs on rat liver mitochondria (RLM) and the human hepatoblastoma cell line (HepG2). HepG2 was selected as a model system due to its known vulnerability to mitochondrial toxicants and the correlation between hepatoxicity and mitochondrial toxicity [46]. Additionally, they exhibit the Crabtree effect, a phenomenon in which cells switch to OXPHOS for ATP production when glucose is substituted with galactose [47]. HepG2 cells have also been widely used to investigate mitochondria as a target for anticancer agents [48]. Since drugs may induce mitochondrial impairment through multiple mechanisms, multiple in vitro assays are required to gain a deeper understanding of them. The main goal of this study was to compare multiple in vitro assays using different model systems and mitochondrial endpoints to shed light on the mitochondrial bioenergetics effects of the psychotropic drugs included in this study. This investigation will constitute a major contribution to the understanding of mechanisms of drug toxicity and side effects, as well as contributing to the development of adverse outcome pathways. Identification of molecular mechanisms of toxicity is valuable since it can lead to the establishment and validation of surrogate high-throughput methods to reduce animal testing for drug safety studies during drug discovery.

## 2. Materials and Methods

### 2.1. Materials

Compounds with the highest available purity were purchased from Sigma-Aldrich (Dorset, UK). The XFe 96 FluxPaks for the XFe 96 Extracellular Flux Analyzer were procured from Seahorse Biosciences (Agilent Technologies, Cheadle, UK; North Billerica, MA, USA). The source of all other supplements and cell culture media was Fisher Scientific (Loughborough, UK). The rPFO was from Seahorse Bioscience as XF PMP.

### 2.2. Cell Culture

The HepG2 cell lines from the Public Health England European Collection of Cell Cultures (ECACC, Salisbury, UK) were cultured in complete Minimal Essential Medium (EMEM) that was supplemented with 10% fetal bovine serum (FBS), 2 mM L-GlutaMAX, 1% non-essential amino acids (NEAA), 53 U/mL penicillin, and 53 μg/mL streptomycin. Cells were maintained in a humidified atmosphere with 5% CO_2_ at 37 °C. Cells were passaged three times a week and kept for up to four weeks.

### 2.3. Isolation of Rat Liver Mitochondria

Mitochondria were isolated from adult Wistar Rat liver. Rats were euthanized via cervical dislocation or with an overdose of CO_2_ and the liver was rapidly excised and immersed into ice-cold Milli-Q water and then transferred into an isolation buffer containing 1 mM EGTA, 30 mM MOPS, 0.25 M sucrose, 3.5 mM L-cysteine, and 0.1% (*w/v*) BSA, pH 7.4. The liver was homogenized with 10 passes in a loose-fitting homogenizer, followed by 10 passes in a tight-fitting homogenizer, and filtered through muslin. The tissue homogenate was then centrifuged at 1000× *g* for 10 min at 4 °C and the supernatants were further centrifuged at 10,000× *g* for 10 min. The centrifugation steps were repeated, preserving the pellet. The resulting final mitochondrial pellet was re-suspended in buffer solution to produce a protein concentration of 30–40 mg/mL and preserved on ice. Protein concentration was determined using the Bradford method with bovine serum albumin as the standard.

### 2.4. Measurement of Mitochondrial Toxicity Using Seahorse XFe96 Extracellular Flux Analyzer—Acute Extracellular Flux (AEF) Assay in Intact HepG2 Cells

The Extracellular Flux Analyzer 96-well format (XFe96, Seahorse Bioscience) was used to simultaneously measure the oxygen consumption rate (OCR) and the extracellular acidification rate (ECAR) in real-time in HepG2 cells. The XFe96 has two fluorescent sensors in each well which detect changes in oxygen and proton levels in the media. From the OCR profile, the effects of the drugs on several respiration parameters were evaluated, including basal respiration, reserve capacity, ATP production, and proton leak. From the ECAR profile, changes in glycolysis were assessed. The day prior to the assay, the cells were seeded on the XFe96 cell culture plates at 20,000 cells/well in culture medium and kept overnight at 37 °C, 5% CO_2_. Respective XFe96 cartridge plates were hydrated using 200 μL/well of calibrant and kept overnight in a 37 °C, non-CO_2_ incubator.

On the day of the assay, cells in the culture plates were washed with non-buffered, pre-warmed, freshly prepared XF Base DMEM medium, supplemented with 10 mM glucose, 1 mM pyruvate, and 2 mM glutaMAX, and adjusted to pH 7.4. Cells were incubated in 180 µL assay media in a non-CO_2_ incubator at 37 °C for 60 min prior to the experiment. During this incubation period, the mitochondrial stressors oligomycin A, carbonyl cyanide-p-trifluoromethoxyphenylhydrazone (FCCP), and the mixture of rotenone/antimycin A were loaded in ports B, C, and D of the cartridge plates, respectively. High concentrations of test compounds (200-fold final concentration) were prepared in DMSO or water. Then, a serial dilution was performed for a seven-point half-log dilution series and were further diluted 1:10 in assay media. Test compounds were injected in port A and the final DMSO concentration was 0.5% (*v/v*) in all wells. On each plate, ten wells were cell-free, six of which were used as compound controls, where the top concentrations of each compound were injected to identify interference with the measurements due to pH changes or color. The remaining four cell-free wells were used as temperature controls.

At the start of each experiment, four initial baseline OCR and ECAR measurements were taken, prior to the addition of the test compounds. Subsequently, test compounds were injected from port A and a further six measurements of OCR and ECAR were taken. This was followed by the injection of oligomycin A (1 μM), an ATP synthase inhibitor, where further two measurements of OCR and ECAR were taken. Another two measurements were taken upon the addition of each of the other mitochondrial stressors, carbonyl cyanide 4-(trifluoromethoxy) phenylhydrazone (0.5 μM FCCP) and then the mixture of ETC inhibitors rotenone and antimycin A, both at 1 μM. Each measurement followed a three-minute mixing and a four-minute reading time.

The following parameters were obtained from OCR readings. (1) Basal OCR was the sixth OCR measurement following compound or vehicle addition, normalized to the baseline OCR measurements. (2) Reserve capacity was calculated from the difference between the basal and the maximal rate of respiration and indicates the capability of the cell to respond to increased energy demand as well as how closely the cell is to respire to its theoretical maximum. (3) ATP production represents the portion of basal respiration that is being used to drive ATP production and was measured upon injection of the ATP synthase inhibitor oligomycin. (4) Proton leak shows the remaining basal respiration not coupled to ATP production. It was calculated from the difference between the respiration rate upon the addition of oligomycin and the non-mitochondrial respiration. All measurements were determined as a change from baseline OCR and corrected for the non-mitochondrial OCR, which is obtained from the final OCR measurement following the addition of reagents rotenone/antimycin A. ECAR measurements were also recorded after the addition of test compounds and normalized to the baseline ECAR. For each compound, graphs of drug concentration vs. response (ECAR and OCR-derived parameter) were drawn. Effects of drugs were assessed by the minimum effective concentration (MEC) that significantly crosses the vehicle control threshold and the concentration at which 50% maximum effect is observed (AC_50_).

### 2.5. Assessing Mitochondrial Respiratory Complexes Using the Seahorse XF Analyzer—Permeabilized HepG2 Cells

The day prior to the assay, HepG2 cells were seeded on the Seahorse Bioscience XFe96 cell culture plates with 20,000 cells/well in culture medium and kept overnight at 37 °C, 5% CO_2_. Respective XFe96 cartridge plates were hydrated with 200 μL/well of calibrant and kept in a 37 °C, non-CO_2_ incubator overnight. In this protocol, 4 nM of recombinant perfringolysin O (rPFO^C459A^) was used to selectively permeabilize the plasma membrane of the cells just before running the assay. On the day of the assay, the test compounds and specific substrates/inhibitors were loaded into the different injection ports of the cartridges. Test compounds were prepared at a 200-fold final concentration in the appropriate vehicle, serially diluted to allow a seven-point half-log dilution series, and were diluted again 1:10 in MAS buffer (220 mM mannitol, 70 mM sucrose, 10 mM KH_2_PO_4_, 5 mM MgCl_2_, 3 mM HEPES, 1 mM EGTA). Test compounds were injected from port A with a total of three technical repeats per assay per concentration. Port B was used for the injection of 10 mM succinate + 2 µM rotenone in MAS buffer. Port C was used for the injection of 10 mM ascorbate + 100 µM TMPD + 2 µM Antimycin A in MAS buffer. Upon calibration of the cartridge, the cell plate was removed from the incubator and was washed once quickly with 0.15 mL MAS buffer + 0.2% BSA (*w/v*). Then, the media was replaced with warm MAS buffer + 0.2% BSA (*w/v*) supplemented with 1 mM malate, 10 mM pyruvate, 4 mM ADP, and 4 nM rPFO, adjusted to pH 7.4 to a final volume of 0.18 mL per well. Immediately after media replacement, the cell plate was inserted into the XF instrument to start the assay.

### 2.6. Assessment of Mitochondrial Toxicity Using Glucose and Galactose Media Conditions (Glu/Gal Assay)

HepG2 cells were collected by trypsinization and 6000 cells/well were plated in a 384-well black microplate and incubated overnight at 37 °C and 5% CO_2_ in DMEM containing 25 mM glucose, 1 mM sodium pyruvate, 10% FCS (*v/v*), and 2 mM glutamine. Six hours prior to the compound treatment, cells were washed with DMEM media containing 1 mM pyruvate, 6 mM glutamine, 10% FBS, 1% NEAA with either glucose (25 mM) or galactose (10 mM). Compounds were prepared at a 200-fold final concentration in 0.5% *v/v* DMSO, except for metformin, valproic acid, and vigabatrin, which were prepared at a 5-fold concentration in the appropriate assay media. This then followed a serial dilution of compounds in the appropriate vehicle to produce an eight-point concentration curve using a half-log dilution series. DMSO dosing solutions were prepared by diluting the compound stocks 1:40 in the glucose or galactose assay media. Cells were then dosed with the Bravo automated liquid handling platform (Agilent Technologies, Santa Clara, CA, USA) using a 1:5 dilution and incubated with compounds for 24 h in a humidified atmosphere with 5% CO_2_ at 37 °C. After this incubation period, cellular ATP was measured using the CellTiter-Glo Cell Viability Assay (Promega Corporation, Madison, USA) as per the manufacturer’s instructions.

### 2.7. High-Resolution Respirometry and Mitochondrial Membrane Potential (MMP)

The Oroboros^®^ Oxygraph-2K (Oroboros Instruments, Innsbruck, Austria [49]) was used to simultaneously analyze high-resolution respirometry and mitochondrial membrane potential (MMP). MMP was measured using safranin, a lipophilic cationic fluorescent probe (Ex/Em = 495/587 nm) that accumulates within the matrix of energized mitochondria. At the beginning of each experiment, the O2k chambers were filled with 2 mL of reaction buffer (200 mM sucrose, 25 mM KCl, 5 mM MgCl_2_, 4 mM KH_2_PO_4_, 5 mM MOPS, pH 7.4) and equilibrated with air at 37 °C. During the experiments, the incubation chambers were kept at constant temperature by Peltier control (±0.001 °C) and the medium was stirred using white PVDF-coated stirrers (750 rpm). For calibration, a 200 µM stock solution of safranin dissolved in distilled water was titrated in five steps into the O2k chamber, up to a final safranin concentration of 2 µM. A linear increase in the fluorescence signal was detected, reflecting the concentration of safranin in the chamber. Safranin cannot be used as an indicator of MMP at high concentrations, as it exerts a dose-dependent inhibitory effect on OXPHOS, particularly when CI-linked respiration is examined [50]. To avoid this problem, the CI inhibitor rotenone and the CII-linked substrate succinate were used. Furthermore, the maximum safranin concentration used in the experiments (2 µM) has been shown to not disturb mitochondrial respiration [50]. Upon calibration of the safranin signal, 100–300 μg of freshly isolated rat liver mitochondria (RLM) was injected into the 2-mL O2k chambers, followed by 1 µM rotenone and 12.5 mM succinate. This was followed by serial additions of the drugs to assess their dose-dependent effect on the MMP and mitochondrial respiration. Injection of RLM results in an uptake of safranin and a corresponding decline in the fluorescence signal. Hence, the signal was normalized within the range of a maximum fluorescence, which corresponded to the basal signal elicited upon the addition of RLM, and a minimum, which corresponded to the fluorescence signal upon the addition of succinate, which also corresponds to the membrane potential generated in the mitochondria. For uncoupled respiration and ROS production measurements, 100–300 μg of freshly isolated RLM were injected into the chambers, followed by 12.5 mM succinate, 1 µM rotenone, and 0.25 µM CCCP.

## 3. Results

**Figure 1:**
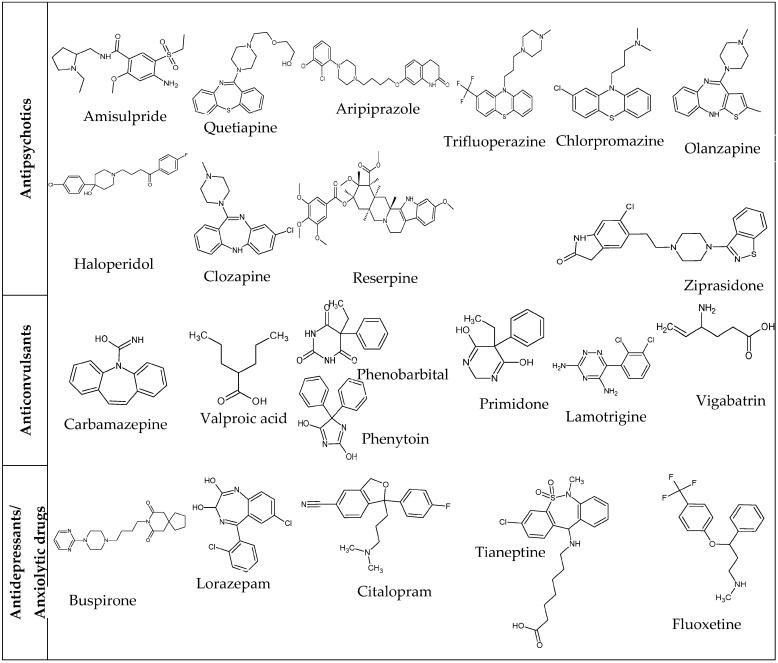
Chemical structures of the psychotropic drugs tested.

A total of 22 psychotropic drugs were included in this study: 10 antipsychotics, 7 anticonvulsants, 3 antidepressants, and 2 anxiolytic drugs. A summary of the drugs’ names and literature mechanisms of mitochondrial toxicity is shown in Table 1 and molecular structures are shown in Figure 1. At the time of writing, a literature search showed mitochondrial liabilities for all these compounds except for amisulpride, lorazepam, and vigabatrin. For the cell assays, the top concentrations tested of each compound were at least 100 × C_max_, the pharmacokinetic parameter indicating the maximum plasma concentration of a drug after a dose, or limit of solubility (Table 2). When possible, literature C_max_ values were obtained for the plasma or serum of adult humans for consistency.

### 3.1. Real-Time Effects of Drugs on Mitochondrial Respiration

**Figure 2:**
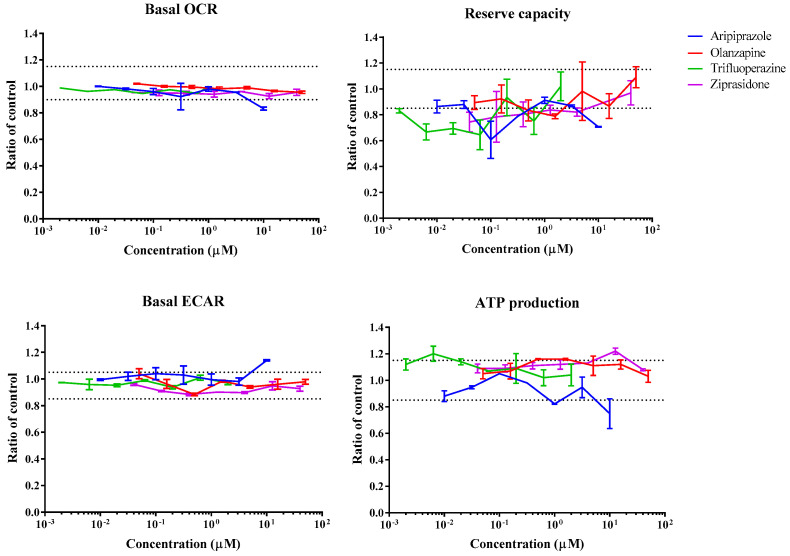
Bioenergetic profile of aripiprazole, olanzapine, trifluoperazine, and ziprasidone using the Extracellular Flux Analyzer. Dose—response curves show the effects of compounds in basal OCR, reserve capacity, basal ECAR, and ATP production in 10 mM glucose, 1 mM pyruvate, and 2 mM glutamine. Data are expressed as a mean ratio to vehicle control ± SD of *n* = 3. Dashed lines represent a significant cut-off from vehicle control.

**Figure 3:**
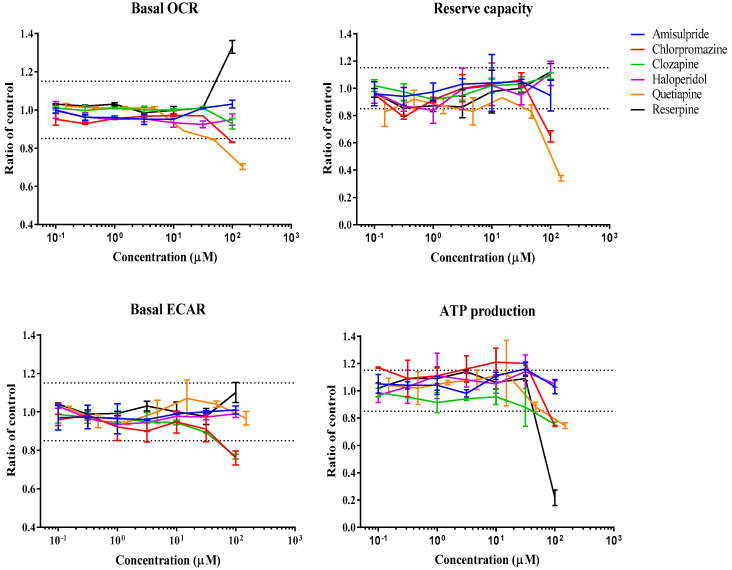
Bioenergetic profile of amisulpride, chlorpromazine, clozapine, haloperidol, quetiapine, and reserpine using the Extracellular Flux Analyzer. Dose—response curves show the effects of compounds in basal OCR, reserve capacity, basal ECAR, and ATP production in 10 mM glucose, 1 mM pyruvate, and 2 mM glutamine. Data are expressed as a mean ratio to vehicle control ± SD of *n* = 3. Dashed lines represent a significant cut-off from vehicle control.

**Figure 4:**
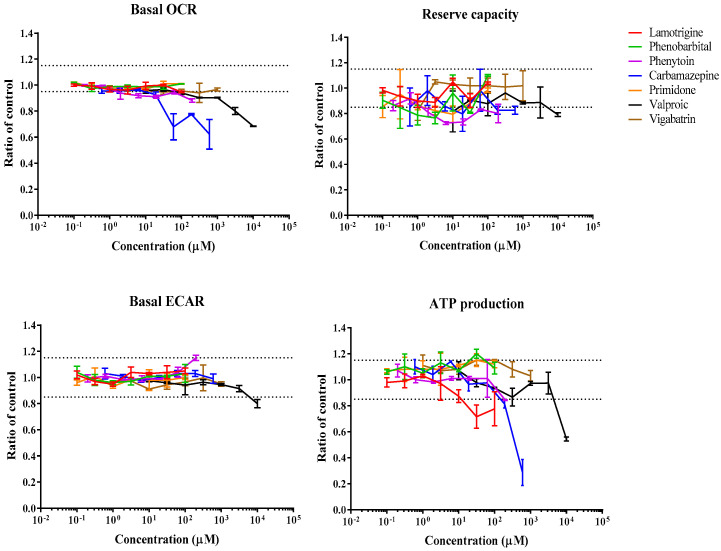
Bioenergetic profile of anticonvulsants tested using the Extracellular Flux Analyzer. Dose—response curves show the effects of compounds in basal OCR, reserve capacity, basal ECAR, and ATP production in 10 mM glucose, 1 mM pyruvate, and 2 mM glutamine. Data are expressed as a mean ratio to vehicle control ± SD of *n* = 3. Black dashed lines represent a significant cut-off from vehicle control.

**Figure 5:**
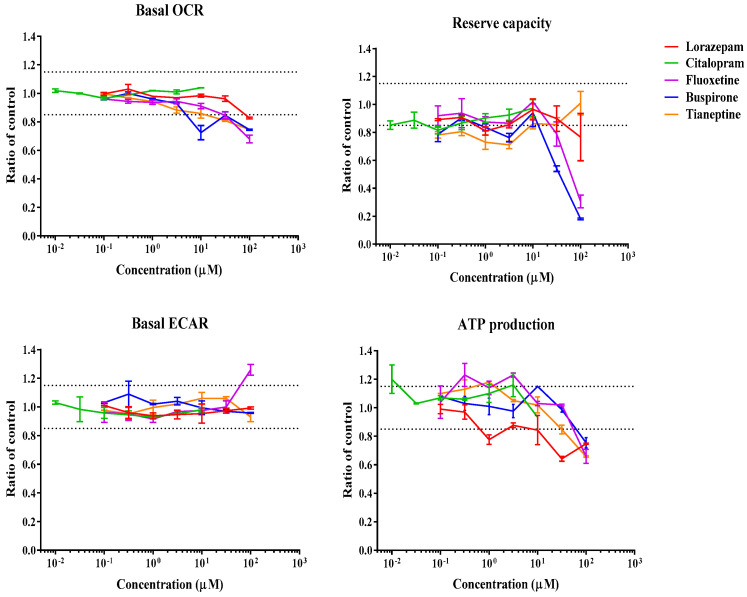
Bioenergetic profile of antidepressants and anxiolytic drugs tested using the Extracellular Flux Analyzer. Dose—response curves show the effects of compounds on basal OCR, reserve capacity, basal ECAR, and ATP production in 10 mM glucose, 1 mM pyruvate, and 2 mM glutamine. Data are expressed as a mean ratio to vehicle control ± SD of *n* = 3. Black dashed lines represent a significant cut-off from vehicle control.

The Seahorse Bioscience XF96 analyzer was used to assess real-time changes in oxygen consumption rate (OCR) and extracellular acidification rate (ECAR) upon injection of drugs onto HepG2 cells. The sequential exposure of cells to various mitochondrial stressors, oligomycin, FCCP, and rotenone/antimycin A, allowed the determination of several mitochondrial parameters, including basal OCR, ATP turnover, reserve capacity or proton leak, in the presence (or absence) of drug concentrations. Figure 2, Figure 3, Figure 4 and Figure 5 show the effect of various drug concentrations on basal OCR, basal ECAR, reserve capacity, and ATP production. Data obtained for all examined drugs and rotenone, a potent CI inhibitor used as the positive control, are summarized in Table 2 and Table 3. This assay allowed the identification of 3 ETC inhibitors, 1 uncoupler, 4 substrate inhibitors, and 2 cytotoxic compounds out of the 22 psychotropic drugs tested (Table 2). ETC inhibitors included aripiprazole, phenytoin, and fluoxetine, which showed a dose-dependent decrease in basal OCR, reserve capacity, and ATP-linked respiration, accompanied by a dose-dependent increase in ECAR, an indirect indicative of glycolysis. In the case of phenytoin, these effects were significant at concentrations close to its C_max_ value (OCR MEC = 90.9 µM, C_max_ = 30 µM) (Table 2 and Table 3). Quetiapine, carbamazepine, buspirone, and tianeptine caused a dose-dependent decrease in OCR, reserve capacity, and ATP production; however, an increase in ECAR was not observed, and therefore they were classified as substrate inhibitors. This could mean that OCR decrease may be due to reduced substrate availability, explained by decreased substrate transport/metabolization mediated by other enzymes, such as the pyruvate dehydrogenase (PDH), the mitochondrial pyruvate carrier (MPC), or the mitochondrial dicarboxylate carrier (DIC). Chlorpromazine and valproic acid caused a drop in both OCR (MEC = 73.4 µM and 520 µM, respectively) and ECAR (MEC = 15.6 µM and 2600 µM, respectively); hence, they were classified as cytotoxic compounds. Reserpine was the only compound that showed an uncoupling activity. This was shown by an increase in OCR (MEC = 60.3 µM) and proton leak (MEC = 30 µM), while mitochondrial ATP production was strongly inhibited (MEC = 59.4 µM). A few compounds were categorized as “other” due to their mode of action being poorly defined using this system (Table 2). For instance, trifluoperazine only caused a small decrease in reserve capacity (MEC < 0.002 µM), while there was no response change in basal OCR or ECAR. Lorazepam decreased basal OCR (MEC = 63.7 µM) and ATP production (MEC = 3.2 µM) and increased proton leak (MEC = 3.14 µM); however, it did not affect ECAR or reserve capacity. Clozapine showed a reduction in ECAR (MEC = 20.2 µM) and ATP (MEC = 37.3 µM), lamotrigine only caused ATP reduction (MEC = 14 µM), citalopram caused a decrease in the proton leak (MEC = 7.17 µM), and ziprasidone showed an increase in ATP (MEC = 0.95 µM) and decrease in the proton leak (MEC = 0.12 µM). Compounds that showed no effect on any of the mitochondrial parameters included amisulpride, haloperidol, olanzapine, phenobarbital, primidone, and vigabatrin (Table 2).

### 3.2. Drugs’ Effects on Respiratory Activity of Permeabilized HepG2 Cells

A combination of rPFO, different substrates, and inhibitors were used to study mitochondrial function in situ. rPFO is a cholesterol-dependent cytolysin derived from Clostridium perfringens that forms oligomeric pores in cholesterol-containing membranes, allowing the passage of small solutes (<200 kDa) through the cell membrane, without affecting mitochondrial membrane permeability [89]. CI-mediated respiratory activity was measured via oxidation of the NADH-linked substrates pyruvate and malate. This was followed by the CII-linked respiration measured with the oxidation of newly added succinate and the addition of CI inhibitor rotenone. Finally, CIV-linked respiration was evaluated with the addition to the media of the non-physiological electron-donating compound, tetramethyl-p-phenylene diamine (TMPD), and the reducing agent, ascorbate (to regenerate the TMPD from its oxidized form), in the presence of CIII-inhibitor, antimycin A.

Compounds that showed a significant response in the previous Acute Extracellular Flux (AEF) assay, i.e., aripiprazole, chlorpromazine, quetiapine, reserpine, carbamazepine, phenytoin, valproic acid, buspirone, fluoxetine, and tianeptine, were investigated here (Table 4). At the beginning of the assay, the compounds were injected onto permeabilized HepG2 cells seeded in media containing CI-linked substrates (1 mM malate and 10 mM pyruvate), 4 mM ADP, and 0.1% fatty acid free bovine serum albumin. Then, changes in OCR with respect to respiration prior to the injection of compounds were recorded. The rest of the assay involved the injection of substrates/inhibitors for the determination of the effects of the compounds on the CII-linked and CIV-linked respirations.

**Figure 6:**
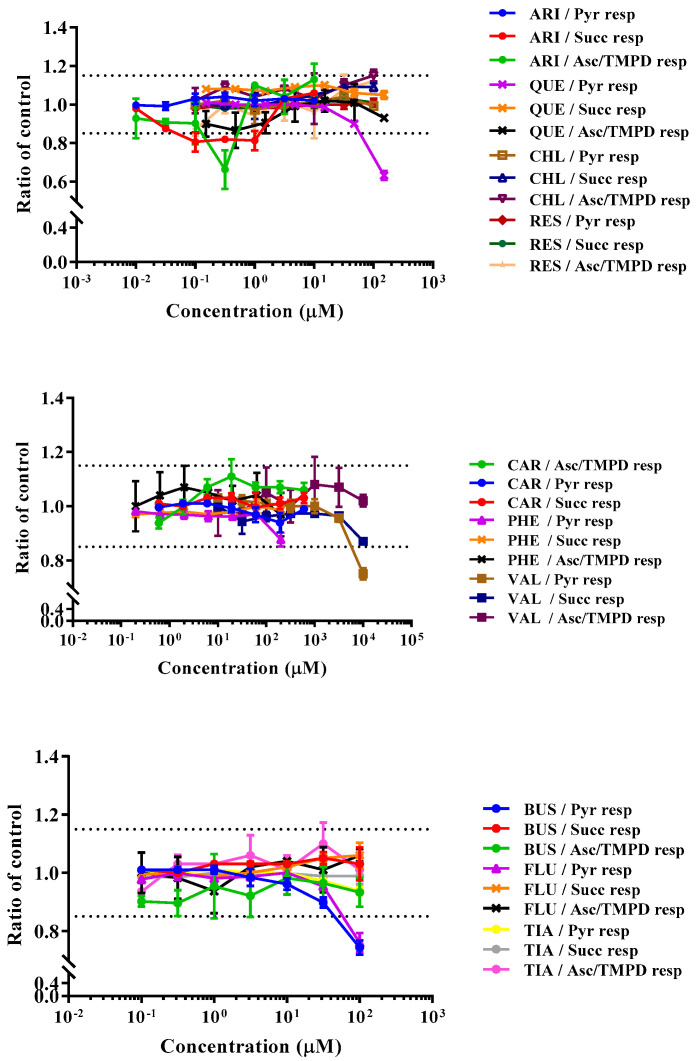
Dose—response curve of compounds in the permeabilized cell assay: pyruvate (pyr) respiration, succinate (succ) respiration and ascorbate/TMPD (asc/TMPD) respiration. ARI = aripiprazole, QUE = quetiapine, CHL = chlorpromazine, RES = reserpine, CAR = carbamazepine, PHE = phenytoin, VAL = valproic acid, BUS = buspirone, FLU = fluoxetine and TIA = tianeptine. Data are expressed as a mean ratio to vehicle control ± SD of *n* = 3.

Among the APs tested, quetiapine, previously categorized in the AEF assay as a substrate inhibitor, showed a significant response inhibiting CI-linked respiration (MEC = 66.3 µM) (Figure 6). Mitochondrial respiration was restored upon the addition of the CII substrate, succinate, indicating that quetiapine may act as an inhibitor of substrates that support CI respiration. Aripiprazole, chlorpromazine, and reserpine, previously categorized as an ETC inhibitor, a cytotoxic compound, and as an uncoupler, respectively (Table 2), did not show a significant response at the concentrations tested here (Figure 6). Among the ACs, phenytoin, carbamazepine, and valproic acid were previously categorized as an ETC inhibitor, a substrate inhibitor, and a cytotoxic compound, respectively (Table 2). Here, only valproic acid showed a significant response, causing inhibition of CI-linked respiration (MEC = 6370 µM) that was restored upon the addition of CII substrate succinate (Figure 6). Among the antidepressant and anxiolytic drugs, buspirone and tianeptine were previously categorized as substrate inhibitors, while fluoxetine was categorized as an ECT inhibitor. Here, the most sensitive mechanism for buspirone and fluoxetine was CI-linked respiration (MEC = 44.5 µM and 72.1 µM, respectively), while tianeptine did not show a significant response at the concentrations tested (0.1–100 µM) (Figure 6). The control compound rotenone showed a strong inhibition of CI-linked respiration (MEC = 0.0009 µM) (Table 4).

### 3.3. Assessment of Mitochondrial Toxicity Using Selective Media Conditions (Glu/Gal Assay)

Mitochondrial toxicity was assessed in HepG2 cells cultured in galactose (Gal) or glucose (Glu) containing media. Cellular ATP levels were measured 24 h after compound treatment to determine cell viability in both media conditions. This assay is based on the increased susceptibility of glycolytic cells to mitochondrial toxicants when forced to rely on mitochondrial OXPHOS for energy production in galactose media (lacking glucose) [90]. MEC and AC_50_ values obtained for drug-induced changes in ATP production in both media conditions are shown in Table 5. Rotenone, used as a positive control, showed a 4600-fold difference in the AC_50_ values between the two media conditions (Table 5). According to Eakins et al. [90], a ≥3-fold shift in AC_50_ values provides the most predictive cut-off for identifying the mitochondrial toxicants, with a sensitivity of 51%, specificity of 97%, and overall accuracy of 72%.

Among the APs tested, 100 μM amisulpride did not show any effect on ATP production in any of the media conditions. Compounds that showed similar toxicity in both media conditions included chlorpromazine, clozapine (100 μM top concentration), quetiapine (150 μM top concentration), and trifluoperazine (2 μM top concentration). Compounds that showed slightly higher toxicity in Glu media included haloperidol (100 μM top concentration) and reserpine (100 μM top concentration). Aripiprazole and ziprasidone showed higher toxicity in Gal media (10 μM and 40 μM top concentration) (Table 5). Ziprasidone was non-responsive in the AEF assay; therefore, the toxicity observed in this current assay could be due to the incubation period. Among the ACs, carbamazepine showed an MEC = 408 μM in cells cultured in Gal media (Table 5). Amongst the antidepressants/anxiolytic drugs, fluoxetine and citalopram showed toxicity trends in both media conditions. In the AEF assay, fluoxetine was identified as an ETC inhibitor; however, here, fluoxetine showed slightly stronger cytotoxicity in Glu medium than Gal medium, with a Glu/Gal AC_50_ ratio of 0.76 (Table 5). This indicates that mitochondrial toxicity may not be fluoxetine’s primary mechanism of toxicity. Finally, buspirone and tianeptine showed reduced cell viability only in Gal media (MEC = 35.7 and 41.8 µM, respectively), suggesting mitochondrial toxicity as their primary mechanism of toxicity (Table 5).

### 3.4. High-Resolution Respirometry Readings

**Figure 7:**
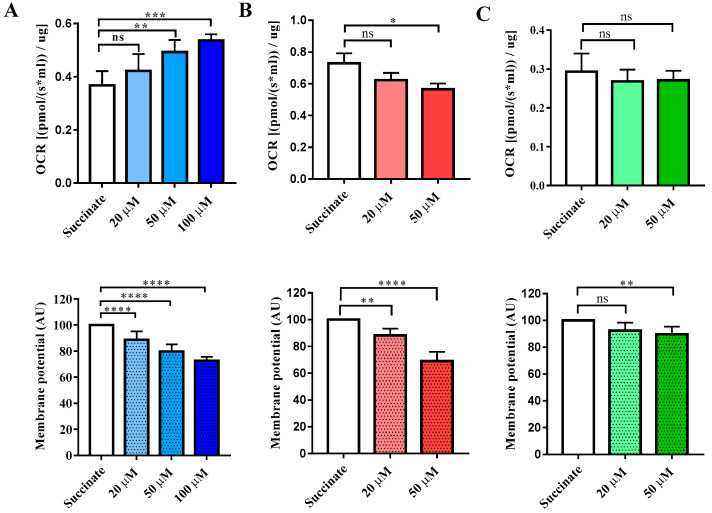
Simultaneous evaluation of OCR normalized to protein content and MMP in freshly isolated RLM in the presence of (**A**) chlorpromazine (blue) (**B**) haloperidol (red) and (**C**) olanzapine (green). Data are mean ± SD of n = 3–6 independent experiments (biological repeats). For the establishment of significance, one-way ANOVA was performed followed by the Dunnet test. Statistically significant values compared with the control are reported as follows: * *p* < 0.05, ** *p* < 0.01, *** *p* < 0.001, **** *p* < 0.0001, and ns: not significant.

The effects of chlorpromazine, haloperidol, and olanzapine were further investigated in freshly isolated rat liver mitochondria, which were used to simultaneously analyze high-resolution respirometry (Oroboros^®^ Oxygraph-2K), changes in mitochondrial membrane potential (MMP), and reactive oxygen species (ROS) production. Upon initial calibration with 2 µM safranin, rat liver mitochondria (RLM), 1 µM rotenone, and 12.5 mM succinate were added to the Oroboros^®^ chambers to induce CII-linked respiration. Then, increasing drug concentrations were titrated into the chambers. The three drugs displayed very different effects on respiration and MMP. Chlorpromazine behaved as an uncoupler of OXPHOS, as it increased the respiratory rate to 111% at 20 μM, to 125% at 50 μM, and to 131% at 100 μM, while it significantly dissipated the MMP to 88% at 20 μM, to 79% at 50 μM, and to 72% at 100 μM (normalized to control, i.e., succinate respiration) (Figure 7). Haloperidol, on the other hand, decreased respiration to 85% at 20 μM and 77% at 50 μM and dissipated the MMP to 88% at 20 μM and to 69% at 50 μM. Olanzapine did not affect respiration but caused a mild dissipation of the MMP at 50 μM (Figure 7).

**Figure 8:**
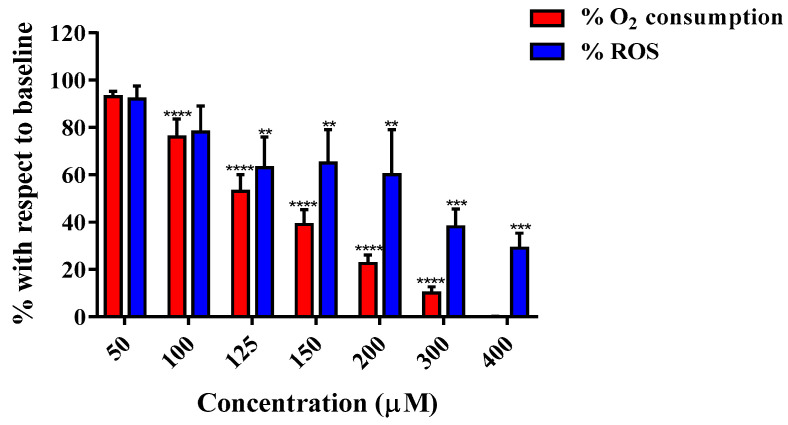
Chlorpromazine effects on ROS production and O_2_ consumption in isolated RLM (uncoupled respiration). Data are mean ± SD of at least 3 independent experiments. ** *p* < 0.01, *** *p* < 0.001, **** *p* < 0.0001 vs. control (succinate respiration).

Chlorpromazine’s effects on RLM mitochondria were investigated further by the simultaneous measurement of uncoupled respiration and ROS production using the Oroboros^®^ Oxygraph-2K. Here, respiration was initiated with 12.5 mM succinate + 1 µM rotenone and stimulated with 0.25 µM CCCP. In contrast to what was observed in basal respiration (not stimulated by CCCP), chlorpromazine reduced the uncoupled respiration in a dose-dependent manner (50–400 µM) with an IC_50_ of 135 ± 5 µM. Note the OCR in Figure 8 is uncoupled, hence it is ‘reserve capacity’, and different from basal OCR depicted in Figure 7. Likewise, increasing concentrations of chlorpromazine led to a reduction in the production of ROS (Figure 8). Both O_2_ consumption and ROS production values are expressed as a percentage with respect to the baseline (succinate respiration).

## 4. Discussion

There is a growing body of evidence that links the use of psychotropic drugs with mitochondrial dysfunction [91]. The present study aimed to shed light on the multiple mechanisms of toxicity linked to mitochondrial dysfunction by a diverse range of psychotropic drugs, including antidepressants, anticonvulsants, antipsychotics, and mood stabilizers, with a focus on ETC inhibition.

### 4.1. Antipsychotics

In this study, three typical antipsychotics (APs) were investigated: chlorpromazine, haloperidol, and trifluoperazine. Chlorpromazine and haloperidol have long been reported as inhibitors of the respiratory CI [92,93,94], which has been suggested to correlate with extrapyramidal side effects. This hypothesis has been supported by studies performed on both rat brain cortex [95] and human brain cortex [96], which showed that haloperidol was a CI inhibitor (IC_50_ = 100 μM), followed in potency by chlorpromazine (IC_50_ = 400 μM) [96]. In pig brain mitochondria, both chlorpromazine and haloperidol inhibited CI-linked respiration with a similar trend (IC_50_s of 64.9 and 116 μM for haloperidol and chlorpromazine respectively), while they were weaker inhibitors of CII-linked respiration [59]. Reduced mitochondrial bioenergetics has also been associated with their reproductive toxicity, where both drugs decreased ATP levels, oxygen consumption rates, and mitochondrial membrane potential in rat ovarian theca cells [20]. In this study, investigations using isolated rat liver mitochondria revealed a dual activity of chlorpromazine depending on the state of respiration. In CII-linked basal respiration, increasing concentrations, from 20 to 100 µM, led to a significant increase in OCR (Figure 7) with a simultaneous dissipation of the MMP, which would suggest an uncoupling activity. However, in CII-linked uncoupled respiration (induced by CCCP injection), a dose-dependent decrease in OCR was observed with an IC_50_ of 135 ± 5 µM (Figure 8). Nevertheless, the acute extracellular flux (AEF) assay in HepG2 cells showed a simultaneous reduction in both OCR and ECAR. Furthermore, the Glu/Gal assay showed reduced cell viability in both culture media conditions at similar AC_50_ values, an indicator of general cytotoxicity (Table 6). Therefore, although results obtained using isolated mitochondria show chlorpromazine can act as an inhibitor or as an uncoupler of the ETC, cell-based assays indicate the existence of other off-mitochondrial target effects which may have a more pronounced contribution to the toxicity of this drug. Haloperidol (100 µM) showed no significant effects on any of the mitochondrial parameters in intact HepG2 cells (Table 6). However, high-resolution respirometry performed using isolated mitochondria showed inhibition of CII-linked respiration and dissipation of the MMP in a dose-dependent manner. Finally, for trifluoperazine, at concentrations more than 400-fold, the C_max_ had no significant effect on mitochondrial OCR in intact HepG2 cells but caused a small reduction in reserve capacity (AC_50_ = 0.157 µM) (Table 6). The Glu/Gal assay revealed that 24 h incubation with 2 µM trifluoperazine leads to complete loss of cell viability in both media (Table 5). These results indicate that its cytotoxicity is not related primarily to mitochondria. Nevertheless, previous studies have shown inhibition of ADP-stimulated respiration in isolated rat liver mitochondria (66 µM) [64,97] and inhibition of the cytochrome *bc_1_* and cytochrome *c-aa_3_* segments of the respiratory chain system of porcine liver and skeletal muscle mitochondria by trifluoperazine [63].

The increased risk of metabolic syndrome observed in patients taking some atypical APs has been associated with mitochondrial dysfunction. In this study, seven atypical APs were investigated: amisulpride, aripiprazole, clozapine, olanzapine, quetiapine, reserpine, and ziprasidone. In previous studies using pig brain mitochondria, aripiprazole, ziprasidone, and quetiapine have been found to inhibit CI-linked respiration (IC_50_ values of 13, 188, and 424 μM, respectively), and CII-linked respiration (IC_50_ values of 32, 86, and 49 μM, respectively) [51]. This was supported by spectrophotometric measurements of enzymatic activities for aripiprazole and quetiapine which showed strong inhibition of CI and partial inhibition of CII + III [51]. Here, the AEF assay identified aripiprazole as a mitochondrial inhibitor of HepG2 cells (OCR MEC = 2.06 μM), and the Glu/Gal assay confirmed a higher susceptibility when cells were grown in galactose media (Gal MEC = 5.67 μM) (Table 6). However, we were not able to pinpoint a precise site of action within the ETC through the permeabilized cell assay at the concentrations tested. The AEF identified quetiapine as a substrate inhibitor (OCR MEC = 19 μM), while the permeabilized cell assay showed CI-linked respiration as the most sensitive mechanism (MEC = 66.3 μM), suggesting quetiapine may inhibit CI-linked respiration substrates. Quetiapine showed a similar cytotoxicity in both Glu and Gal media conditions, suggesting other cellular mechanisms of toxicity (Table 6); nevertheless, given the generally low sensitivity of this assay, this result is not an indicator of an absence of mitochondrial toxicity [90]. Clozapine has been shown to inhibit CI activity in mononuclear cells of patients treated for a minimum of 28 months [94]. Ovaries of rats treated with clozapine (20 mg/kg/day) for 28 days showed a reduction in complex I activity, but not complex III [98]. Spectrophotometric measurements of mitochondrial respiratory complexes from pig brain mitochondria have showed that 30 min incubation with 50 µM clozapine decreased complex I activity by 85% [51]. Here, the AEF assay only detected a small decrease in ECAR and ATP production by 100 µM clozapine, but a precise mode of action could not be identified (Table 6). Clozapine is extensively metabolized in the liver by various CYP450s [99] and bioactivated to a reactive nitrenium ion [100], which could be associated to the mechanisms of clozapine-induced toxicity. Like clozapine, ziprasidone showed an undetermined effect on mitochondrial function through the AEF assay. However, the Glu/Gal assay showed cytotoxicity only in the Gal media (MEC = 28.3 µM) (Table 6), which strongly indicates ziprasidone’s mitochondrial effect. The response observed in the Glu/Gal assay could be due to the incubation period. Incubation time is a major contributor to variability in cell viability assays [101]. The time-dependent effects of enzyme inhibitors can be attributed to metabolism/degradation during incubation time or mechanism-based inhibition [102] Ziprasidone is known to be extensively metabolized in vivo with only a small amount excreted as an unchanged drug. In clinical settings, oxidation by cytochrome P450 and, more importantly, reduction by aldehyde oxidase are the main routes of metabolism [103]. Spectrophotometric measurements of mitochondrial respiratory complexes from pig brain mitochondria have showed that 30 min incubation with 50 µM olanzapine caused no significant effects in any respiratory complex [51], while 500 µM olanzapine inhibited CI and CIV activities, but not CII [54]. Here, 50 µM olanzapine did not cause a decrease in respiration in intact HepG2 cells after acute injection and did not cause a reduction in cellular ATP levels after 24 h incubation treatment in either Glu or Gal media. Only a 10% decrease in the MMP was observed in isolated rat liver mitochondria at 50 µM (Table 6). Reserpine has previously been shown to be an uncoupling agent in isolated monkey liver mitochondria [61]. Here, reserpine showed an acute uncoupling activity in HepG2 by causing a sharp increase in OCR and decreasing ATP production (Figure 3). At the time of writing, amisulpride had no reported mitochondrial effects in the literature and we did not observe any significant effects in any of our in vitro assays (Table 6).

### 4.2. Anticonvulsants

A study using isolated pig brain mitochondria found that 30 min incubation with 50 µM carbamazepine led to significant inhibition of the enzymatic activity of CI and a mild inhibition of CIV, while CII+III activity was not affected. The same study also found that carbamazepine was a partial inhibitor of CI-linked respiration at >100 µM (IC_50_ = 353 µM) and CII-linked respiration (182 µM) in isolated mitochondria [44]. Here, acute injection of carbamazepine onto HepG2 cells showed a decrease in OCR (MEC = 13.1 µM), reserve capacity, ATP production, and proton leak, while ECAR remained unaffected; therefore, it was categorized as a substrate inhibitor. The permeabilized assay did not shed light on the mechanism of action, which could be attributed to carbamazepine’s high protein binding. The Glu/Gal assay confirmed carbamazepine is a mitochondrial toxicant by showing toxicity in Gal media only (Table 7).

A study conducted on lymphocytes of children treated for 24 months with lamotrigine found significantly increased ATP production in comparison to untreated controls, while the enzymatic activities of CII, CII+III, or CIV were not significantly affected [53]. Other studies have shown lamotrigine can partially inhibit both CI- and CII-linked respiration at higher concentrations than tested here (IC_50_s of 332 and 381 µM, respectively) [44]. In this study, lamotrigine only showed a small decrease in ATP production (MEC = 14 µM) (Table 3) in intact HepG2 cells. The Glu/Gal assay showed no cytotoxicity after 24 h incubation with 100 µM lamotrigine in any of the media conditions (Table 7).

More than 95% of phenytoin is biotransformed by the liver and less than 5% is eliminated unchanged in urine [34]. In a murine hepatic microsomal system, the bioactivated phenytoin, but not the parent drug, impaired ATP synthesis at 50 µM and intensively affected mitochondrial respiration, including state 3 respiration (decreased at 200 µM), the respiratory control ratio (decreased at 50 µM), and state four respiration (increased at 50 µM) [41]. Here, phenytoin decreased OCR, reserve capacity, and ATP production, while it increased ECAR in intact HepG2 cells; therefore, it was categorized as an ETC inhibitor (Table 7). However, the cell permeabilized and the Glu/Gal assay did not reveal any mechanistic information or show any toxicity.

Valproic acid is a widely used antiepileptic drug considered to be the third most common drug suspected of causing death because of hepatotoxicity [104]. Valproic-acid-induced mitochondrial toxicity has been extensively documented, particularly in patients with mitochondrial disorders; however, the exact mechanisms leading to liver injury are still unclear [105,106,107,108,109]. A study found a statistically significant decrease in CI and CIV activity in isolated pig brain mitochondria after 30 min incubation with 5 mM valproic acid [54]. Liver mitochondria from rats fed with 1% (*w/w*) valproic acid for 75 days displayed a 30% decrease in the respiration rate with substrates feeding CI and CII. The inhibition was found to be located at the site of the proton-pumping activity of complex IV [109]. Additionally, valproic acid has been reported to inhibit mitochondrial fatty acid β-oxidation [65] and to induce the MPTP opening [66]. In this study, acute injection of valproic acid onto HepG2 cells caused a dose-dependent decrease in OCR, reserve capacity, ATP production, and ECAR, and a dose-dependent increase in proton leak (Table 7). The MEC values for these mitochondrial dysfunction effects were close to the clinically relevant concentrations (C_max_ value = 368 µM), making such adverse reactions a practical possibility. The most sensitive mechanism of ETC inhibition for valproic acid in permeabilized HepG2 cells was inhibition of pyruvate respiration (Figure 6), which is in agreement with previous studies [54]. Nevertheless, the reduction in both OCR and ECAR in the AEF assay suggests the existence of other mechanisms by which valproic acid exerts its toxic effects in hepatocytes. Finally, phenobarbital (0.1–100 µM), primidone (0.1–100 µM), and vigabatrin (1–1000 µM) did not show any significant response in any of the in vitro assays at the concentrations tested. In the literature, phenobarbital has been shown to reduce CI-, CII-, and CIV-linked respiration in rat liver mitochondria at 500 µM [41].

### 4.3. Antidepressants and Anxiolytic Drugs

Buspirone is an anxiety medication with limited data in the literature regarding its effects on mitochondrial function. Dykens et al. [52] showed buspirone exhibited cytotoxicity only in Gal media in HepG2 cells (60% depletion at 200 µM), but not in Glu media. Similar results were obtained in our study, where buspirone decreased cellular ATP in Gal media only (AC_50_ = 99 µM) (Table 8). Dykens et al. [52] also reported OCR inhibition accompanied by ECAR increase in HepG2 cells at 100 µM. In our study, we observed a decrease in OCR (AC_50_ > 100 µM); however, an increase in ECAR was not detected and buspirone was categorized as a substrate inhibitor using our system (Table 8). In addition, in agreement with our permeabilized cell assay results, Dykens et al. [52] showed inhibition of CI (IC_50_ = 48 µM), but not CII/III, CIV, or CV in isolated mitochondria. Here, the permeabilized HepG2 cell assay revealed that the most sensitive mechanism for buspirone was the inhibition of pyruvate respiration (Table 8, Figure 6). This latter finding, along with the results from Dykens et al., suggest inhibition of CI-linked respiration to be the primary mechanism of mitochondrial inhibition, and that our initial AEF assay may have lacked sensitivity to detect ECAR increase.

A literature search on the effects of lorazepam on mitochondrial function did not report significant findings at the time of writing. In this study, we report for the first time that lorazepam caused a significant decrease in OCR (MEC = 63.7 µM, AC_50_ > 100 µM) and ATP production (MEC = 3.2 µM, AC_50_ > 100 µM), while ECAR remained unaffected (Table 8), suggesting indirect inhibition of OXPHOS. This is supported by the results of permeabilized cell assay where 100 µM lorazepam did not significantly inhibit any of the ETC complexes (Figure 6). Moreover, 100 µM lorazepam did not exhibit toxicity neither in glucose nor in galactose-grown cells after 24 h incubation (Table 8).

Citalopram and fluoxetine are widely prescribed antidepressants that belong to the serotonin reuptake transporter inhibitor (SSRI) class. There are several reports on toxic effects associated with their use, particularly regarding hepatotoxicity [110,111,112]. In a reference database of drugs published by Chen et al. [113], which was part of the FDA’s Liver Toxicity Knowledge Base (LTKB), citalopram and fluoxetine were categorized as “Less-DILI concern”, meaning warnings have been attributed regarding mild DILI probabilities. The mechanisms by which citalopram causes liver injury are unknown. A study by Ahmadian et al. [111] investigated the mechanisms of citalopram-induced hepatotoxicity in freshly isolated rat hepatocytes, where 500 µM citalopram caused cell death, mitochondrial potential collapse, glutathione depletion, lipid peroxidation, and increased ROS formation. In another study using isolated pig brain mitochondria, 500 µM citalopram significantly inhibited CI activity [54]. Here, the highest concentration tested for citalopram was 10 µM, which is 100-fold its C_max_ (Table 2) value. The concentrations investigated here did not report any significant effect in the AEF assay, except for a reduction in proton leak (MEC = 7.17 µM) (Table 8). The Glu/Gal assay reported a similar reduction in cell viability in both media conditions (around 40% at 10 µM), which suggests mechanisms of toxicity other than mitochondrial (Table 8).

Several studies have previously reported the fluoxetine-induced effects on mitochondrial function. In a study published by Souza et al. [58], fluoxetine inhibited CI-linked respiration by 50% at 250 µM in rat liver isolated mitochondria. The same study also found that fluoxetine inhibited the ATP synthase non-competitively in vitro through direct binding with the membrane F_0_ component, with 50% of the effect at 60 µM. Curti et al. [57] also found that fluoxetine decreased CI-linked respiration (IC_50_ = 150 µM) in isolated rat brain mitochondria. Additionally, fluoxetine decreased the activity of F_1_F_0_-ATPase in submitochondrial particles (IC_50_ = 80 µM). Another study conducted in pig brain mitochondria also showed inhibition of CI-linked respiration (IC_50_ = 86.2 µM) [114]. Our results showed fluoxetine acts as a direct ETC inhibitor in HepG2 cells, causing a decrease in OCR, reserve capacity, and ATP production, accompanied by an increase in ECAR (Table 8). Furthermore, the permeabilized assay supports the literature findings by revealing inhibition of CI-linked respiration as the most sensitive mechanism for fluoxetine-induced mitochondrial toxicity (AC_50_ >100 µM) (Figure 6).

Finally, spectrophotometric measurements revealed close to 50% inhibition of CI activity after 18 h incubation with 50 µM tianeptine in CHO cells, while no inhibition of CII+III or CIV activities was observed following tianeptine treatment [45]. Hroudova et al. [59] measured the inhibitory effects of some antidepressants on the activities of respiratory ETC complexes of isolated pig brain mitochondria and showed tianeptine was the most potent inhibitor of mitochondrial respiration energized through both CI (IC_50_ = 88.9 µM) and CII (IC_50_ = 67.4 µM). Here, acute injection of tianeptine caused a dose-dependent decrease in OCR, reserve capacity, and ATP production in HepG2 cells. Using our system, tianeptine was categorized as a substrate inhibitor due to a lack of effect in the ECAR signal (Table 8). Tianeptine´s mechanism of action was then further investigated in the cell permeabilized assay; however, no significant response was observed. Nevertheless, the Glu/Gal assay revealed glucose-grown cells were resistant to 24 h treatment with 100 µM tianeptine, while galactose-grown cells showed high levels of cytotoxicity (Table 5), indicating that mitochondrial toxicity might be tianeptine’s primary mechanism of cytotoxicity.

## 5. Concluding Remarks

This research has demonstrated the challenges associated with trying to understand why and how drug-induced mitochondrial toxicity occurs. It also highlights the complexities of the design of high-throughput in vitro screening methods for the estimation of toxicities with reasonable accuracy. Parameters such as permeabilized vs. intact cells, acute treatment vs. incubation, the length of incubation, mitochondrial substrates, and mitochondrial state are of paramount importance when capturing drug toxicities that may be relevant to clinical situations. Our results show the limitations of individual high-throughput methods in evaluating the mechanisms of mitochondrial effects of drugs. A true picture of drug interactions and mitochondrial toxic effects will probably only be obtained through the combination of multiple approaches (in vitro, in vivo, and in silico), different model systems, and measurement of multiple mitochondrial endpoints.

Our results, in agreement with previously reported literature, show that a drug can often inhibit multiple pathways that lead to the observed change in respiration. Identifying the exact “molecular initiating event(s)” is essential both for mechanistic understanding of mitochondrial bioenergetics as well as the improved pharmacotherapy outcomes in individual patients. This is a growing area with high potential to affect the healthcare system, which could lead to personalized medicine in the future. Personalized medicine would involve understanding that there is great patient-to-patient variation due to factors such as age, gender, pre-existing conditions, co-medications, state of the immune system, and genetic variants that may play crucial roles in determining susceptibility to drug-induced mitochondrial toxicity. For instance, mitochondrial quality and function declines with age and certain mtDNA haplogroups (distinct patterns in single-nucleotide polymorphisms) are associated with increased individual susceptibility to drug-induced mitochondrial toxicity [115,116,117,118]. In those cases, prescription of certain pharmaceutical drugs with known mitochondrial liabilities should be avoided and alternative safer options should be considered.

## Figures and Tables

**Table 1:** Summary table of compounds tested, drug class, and literature mechanism of action of mitochondrial toxicity. N/A: Not applicable.

Compounds	Drug Class	Literature Mechanism	Reference
Amisulpride	Antipsychotic	no reported effects	N/A
Aripiprazole	Antipsychotic	CI inhibitor	[51]
Buspirone	Anxiolytic	CI inhibitor	[52]
Carbamazepine	Anticonvulsant	decreased ATP production	[53]
		CI and IV inhibitor	[44]
Chlorpromazine	Antipsychotic	CI and IV inhibitor	[20,51]
Citalopram	Antidepressant	CI inhibitor	[54]
		increased ROS, loss of MMP	[55]
Clozapine	Antipsychotic	CI, II+III, and IV inhibitor	[20,51,56]
Fluoxetine	Antidepressant	F_1_F_0_ ATPase inhibitor decreased state 3 respiration and RCR	[57] [58]
		decreased CI and CII-linked respiration	[59]
Haloperidol	Antipsychotic	CI inhibitor	[51]
Lamotrigine	Anticonvulsant	increased ATP production	[53]
		decreased CI-linked respiration	[44]
Lorazepam	Anxiolytic	no reported effects	N/A
Olanzapine	Antipsychotic	CI and IV inhibitor	[54]
		activation of citrate synthase activity	[54]
Phenobarbital	Anticonvulsant	Decreased CI, II, and IV-linked respiration	[41]
Phenytoin (bioactivated)	Anticonvulsant	decreased state-3 respiration and ATP synthesis	[41]
Primidone	Anticonvulsant	enhanced SOD activity and decrease in monoamine oxidases	[60]
Quetiapine	Antipsychotic	CI inhibitor	[51]
Reserpine	Antipsychotic	uncoupler	[61]
Tianeptine	Antidepressant	mitochondrial FAO inhibitor	[62]
		CI and II inhibitor	[54]
Trifluoperazine	Antipsychotic	ETC inhibitor	[63]
		ATPase inhibitor	[64]
Valproic acid	Anticonvulsant	CI and IV inhibitor	[54]
		mitochondrial FAO inhibitor	[65]
		MPTP opening	[66]
		decreased OCR, MMP, ATP, and increased ROS	[67]
Vigabatrin	Anticonvulsant	no reported effects	N/A
Ziprasidone	Antipsychotic	CII, III, and IV inhibitors	[19,51]

**Table 2:** Acute extracellular flux (AEF) assay for the detection of mitochondrial toxicity. List of drugs, literature C_max_ values, range of concentrations tested, and direction of change in basal OCR, reserve capacity, basal ECAR, ATP production, and proton leak, where ↑ = increase compared to vehicle control, ↓ = decrease compared to vehicle control, NR = no response compared to vehicle control. N/A = not available. The summary mechanism is the conclusion made based on the direction of change in the bioenergetic parameters.

Compounds	C_max_ µM	C_max_ Reference	Conc. Range µM	Direction of Change					Summary Mechanism
				OCR	Reserve Capacity	ECAR	ATP	Proton Leak	
Amisulpride	2.56	[68]	0.1–100	NR	NR	NR	NR	NR	-
Aripiprazole	0.7	[69]	0.01–10	↓	↓	↑	↓	NR	ETC inhibitor
Chlorpromazine	0.9	[70]	0.1–100	↓	↓	↓	↓	↑	Cytotoxicity
Clozapine	0.22	[71]	0.1–100	NR	NR	↓	↓	NR	Other
Haloperidol	0.02	[72]	0.1–100	NR	NR	NR	NR	NR	-
Olanzapine	0.02	[73]	0.05–50	NR	NR	NR	NR	NR	-
Quetiapine	1.88	[74]	0.15–150	↓	↓	NR	↓	NR	Substrate inhibitor
Reserpine	0.0004	[75]	0.1–100	↑	NR	NR	↓	↑	Uncoupler
Trifluoperazine	0.005	[76]	0.002–2	NR	↓	NR	NR	NR	Other
Ziprasidone	0.11	[77]	0.04–40	NR	NR	NR	↑	↓	Other
Carbamazepine	6.3	[78]	0.6–600	↓	↓	NR	↓	↓	Substrate inhibitor
Lamotrigine	4	[79]	0.1–100	NR	NR	NR	↓	NR	Other
Phenobarbital	13.8	[80]	0.1–100	NR	NR	NR	NR	NR	-
Phenytoin	30	[81]	0.2–200	↓	↓	↑	↓	NR	ETC inhibitor
Primidone	N/A	N/A	0.1–100	NR	NR	NR	NR	NR	-
Valproic acid	367	[82]	10–10,000	↓	↓	↓	↓	↑	Cytotoxicity
Vigabatrin	4	[83]	1–1000	NR	NR	NR	NR	NR	-
Buspirone	0.003	[84]	0.1–100	↓	↓	NR	↓	NR	Substrate inhibitor
Lorazepam	0.1	[85]	0.1–100	↓	NR	NR	↓	↑	Other
Citalopram	0.1	[86]	0.01–10	NR	NR	NR	NR	↓	Other
Fluoxetine	0.04	[87]	0.1–100	↓	↓	↑	↓	NR	ETC inhibitor
Tianeptine	0.62	[88]	0.1–100	↓	↓	NR	↓	NR	Substrate inhibitor
Rotenone	N/A	N/A	0.003–1	↓	↓	↑	↓	↓	ETC inhibitor

**Table 3:** Data summary of acute extracellular flux assay. MEC = minimum effective concentration that significantly crosses the vehicle control threshold. AC_50_ = the concentration at which a 50% maximum effect is observed. NR = no response observed.

Compounds	MEC (µM)					AC_50_ (µM)				
	OCR	Reserve Capacity	ECAR	ATP Production	Proton Leak	OCR	Reserve Capacity	ECAR	ATP Production	Proton Leak
Amisulpride	NR	NR	NR	NR	NR	NR	NR	NR	NR	NR
Aripiprazole	2.06	0.344	9.20	3.49	NR	>10	>10	>10	>10	NR
Chlorpromazine	73.4	78.4	15.6	94.2	31	>100	>100	>100	>100	>100
Clozapine	NR	NR	20.2	37.3	NR	NR	NR	>100	>100	NR
Haloperidol	NR	NR	NR	NR	NR	NR	NR	NR	NR	NR
Olanzapine	NR	NR	NR	NR	NR	NR	NR	NR	NR	NR
Quetiapine	19	26.1	NR	92.3	NR	>150	124	NR	>150	NR
Reserpine	60.3	NR	NR	59.4	30	>100	NR	NR	77.3	36.8
Trifluoperazine	NR	<0.002	NR	NR	NR	NR	0.157	NR	NR	NR
Ziprasidone	NR	NR	NR	0.949	0.12	NR	NR	NR	>12.7	2.19
Carbamazepine	13.1	137	NR	165	97	>600	>190	NR	363	>600
Lamotrigine	NR	NR	NR	14	NR	NR	NR	NR	75.7	NR
Phenobarbital	NR	NR	NR	NR	NR	NR	NR	NR	NR	NR
Phenytoin	90.9	1.45	138	197	NR	>200	26.6	>200	>200	NR
Primidone	NR	NR	NR	NR	NR	NR	NR	NR	NR	NR
Valproic acid	520	3090	2600	5200	1140	>10,000	>10,000	>10,000	>10,000	9180
Vigabatrin	NR	NR	NR	NR	NR	NR	NR	NR	NR	NR
Buspirone	4.83	8.57	NR	80.9	NR	>100	43.7	NR	>100	NR
Lorazepam	63.7	NR	NR	3.2	3.14	>100	NR	NR	>100	15
Citalopram	NR	NR	NR	NR	7.17	NR	NR	NR	NR	>10
Fluoxetine	8.12	25.2	67.3	68.6	NR	>100	70.4	>100	>100	NR
Tianeptine	5.5	0.182	NR	40.3	NR	>100	21.6	NR	>100	NR
Rotenone	0.0053	<0.003	0.0274	0.0159	0.0120	0.0433	0.0106	>1	0.0459	0.213

**Table 4:** Summary table of the Permeabilized assay. AC_50_ = the concentration at which 50% maximum effect is observed. MEC = minimum effective concentration that significantly crosses vehicle control threshold, ⮁ = direction of response, NR = no response observed.

Compounds	rPFO Permeabilized HepG2 Cells							Intact HepG2 Cells
	⮁	Pyruvate Respiration (μM)		Succinate Respiration (μM)		Ascorbate Respiration (μM)		Outcome of AEF Assay on Intact HepG2 Cells
		AC_50_	MEC	AC_50_	MEC	AC_50_	MEC	
Aripiprazole		NR	NR	NR	NR	NR	NR	ETC inhibitor
Chlorpromazine		NR	NR	NR	NR	NR	NR	Cytotoxicity
Quetiapine	↓	>150	66.3	NR	NR	NR	NR	Substrate inhibitor
Reserpine		NR	NR	NR	NR	NR	NR	Uncoupler
Carbamazepine		NR	NR	NR	NR	NR	NR	Substrate inhibitor
Phenytoin		NR	NR	NR	NR	NR	NR	ETC inhibitor
Valproic acid	↓	>10000	6730	NR	NR	NR	NR	Cytotoxicity
Buspirone	↓	>100	44.5	NR	NR	NR	NR	Substrate inhibitor
Fluoxetine	↓	>100	72.1	NR	NR	NR	NR	ETC inhibitor
Tianeptine		NR	NR	NR	NR	NR	NR	Substrate inhibitor
Rotenone	↓	0.0071	0.0009	NR	NR	NR	NR	ETC inhibitor

**Table 5:** Summary table of the Glu/Gal assay. AC_50_ = the concentration at which a 50% maximum effect on cellular ATP production is observed. MEC = minimum effective concentration that significantly crosses vehicle control threshold, ⮁ = direction of response change. NR = no response observed. UD = undetermined toxicity due to the lack of an AC_50_ value.

	Compounds	⮁	Glu AC_50_ (μM)	Glu MEC (μM)	⮁	Gal AC_50_ (μM)	Gal MEC (μM)	AC_50_ Fold Change
Antipsychotics	Amisulpride		NR	NR		NR	NR	NR
	Aripiprazole		NR	NR	↓	9.17	5.67	UD
	Chlorpromazine	↓	15.9	10.7	↓	16.9	10.5	0.941
	Clozapine	↓	56.7	34.1	↓	60.8	44.4	0.933
	Haloperidol	↓	57.5	26.2	↓	78.6	26.0	0.732
	Olanzapine		NR	NR		NR	NR	NR
	Quetiapine	↓	76.7	49.3	↓	79.0	57.7	0.971
	Reserpine	↓	48.9	6.68	↓	79.0	19.0	0.619
	Trifluoperazine	↓	1.0	0.75	↓	1.13	0.80	0.885
	Ziprasidone		NR	NR	↓	>40	28.3	UD
Anticonvulsants	Carbamazepine		NR	NR	↓	>600	408	UD
	Lamotrigine		NR	NR		NR	NR	NR
	Phenobarbital		NR	NR		NR	NR	NR
	Phenytoin		NR	NR		NR	NR	NR
	Primidone		NR	NR		NR	NR	NR
	Valproic acid		NR	NR		NR	NR	NR
	Vigabatrin		NR	NR		NR	NR	NR
Antidepressants/ Anxiolytic drugs	Buspirone		NR	NR	↓	99	35.7	UD
	Lorazepam		NR	NR		NR	NR	NR
	Citalopram	↓	>10	4.43	↓	>10	7.11	UD
	Fluoxetine	↓	13.3	6.89	↓	17.5	12.1	0.76
	Tianeptine		NR	NR	↓	57.6	41.8	UD
CI inhibitor	Rotenone	↓	27.8	0.29	↓	0.00605	0.002	4600

**Table 6:** Summary of the in vitro assays performed to investigate the mitochondrial effects of the antipsychotics. Direction of change in basal OCR, reserve capacity, basal ECAR, ATP production, proton leak and cell viability, where: ↑ = increase compared to vehicle control, ↓ = decrease compared to vehicle control, NR = no response compared to vehicle control. N/A = not available.

Assays		Amisulpride	Aripiprazole	Chlorpromazine	Clozapine	Haloperidol	Olanzapine	Quetiapine	Reserpine	Trifluoperazine	Ziprasidone
Acute HepG2 Extracellular Flux Assay	OCR (AC_50_ μM)	NR	>10↓	>100↓	NR	NR	NR	>150↓	>100↑	NR	NR
	Reserve capacity (AC_50_ μM)	NR	>10↓	>100↓	NR	NR	NR	124↓	NR	0.157↓	NR
	ECAR (AC_50_ μM)	NR	>10↑	>100↓	>100↓	NR	NR	NR	NR	NR	NR
	ATP production (AC_50_ μM)	NR	>10↓	>100↓	>100↓	NR	NR	>150↓	77.3↓	NR	>12.7↑
	Proton leak (AC_50_ μM)	NR	NR	>100↑	NR	NR	NR	NR	36.8↑	NR	2.19↓
	Summary mechanism	NR	ETC inhibitor	Cytotoxicity	Other	NR	NR	Substrate inhibitor	Uncoupler	Other	Other
Permeabilized HepG2 Extracellular Flux Assay (OCR)	Most sensitive mechanism (AC_50_ μM)	N/A	NR	NR	N/A	N/A	N/A	Pyruvate respiration↓ >150	NR	N/A	N/A
Glu/Gal assay (cell viability reduction, 24 h incubation treatment)	Glucose (AC_50_ μM)	NR	NR	15.9↓	56.7↓	57.5↓	NR	76.7↓	48.9↓	1↓	NR
	Galactose (AC_50_ μM)	NR	9.17↓	16.9↓	60.8↓	78.6↓	NR	79↓	79↓	1.13↓	>40↓
	Fold change	NR	UD	0.941	0.933	0.732	NR	0.971	0.619	0.885	UD
Basal succinate-driven respiration in isolated RLM	% MMP (Conc. μM)			88 (20), 79 (50), 72 (100)		88 (20), 69 (50)	92 (20), 90 (50)				
	% O_2_ consumption (Conc. μM)			111 (20), 125 (50), 131 (100)		85 (20), 77 (50)	90 (20), 92 (50)				
C_max_ (μM)		2.56	0.7	0.9	0.22	0.02	0.02	1.88	0.0004	0.005	0.11

**Table 7:** Summary of the in vitro assays performed to investigate the mitochondrial effects of the anticonvulsants. Direction of change in basal OCR, reserve capacity, basal ECAR, ATP production and proton leak, where: ↑ = increase compared to vehicle control, ↓ = decrease compared to vehicle control, NR = no response compared to vehicle control. N/A = not available.

Assays		Carbamazepine	Lamotrigine	Phenobarbital	Phenytoin	Primidone	Valproic Acid	Vigabatrin
Acute HepG2 Extracellular Flux Assay	OCR (AC_50_ μM)	>600↓	NR	NR	>200↓	NR	>10,000↓	NR
	Reserve capacity (AC_50_ μM)	>190↓	NR	NR	26.6↓	NR	>10,000↓	NR
	ECAR (AC_50_ μM)	NR	NR	NR	>200↑	NR	>10,000↓	NR
	ATP production (AC_50_ μM)	363↓	75.7↓	NR	>200↓	NR	>10,000↓	NR
	Proton leak (AC_50_ μM)	>600↓	NR	NR	NR	NR	9180↑	NR
	Summary mechanism	Substrate inhibitor	Other	NR	ETC inhibitor	NR	Cytotoxicity	NR
Permeabilized HepG2 Extracellular Flux Assay (OCR)	Most sensitive mechanism (AC_50_ μM)	NR	N/A	N/A	NR	N/A	pyruvate respiration↓ >10,000	N/A
Glu/Gal assay (cell viability reduction, 24 h incubation treatment)	Glucose (AC_50_ μM)	NR	NR	NR	NR	NR	NR	NR
	Galactose (AC_50_ μM)	>600↓	NR	NR	NR	NR	NR	NR
	Fold Change	UD	NR	NR	NR	NR	NR	NR
C_max_ (μM)		6	4	89	30	N/A	367	4

**Table 8:** Summary of in vitro assays performed to investigate the mitochondrial effects of the antidepressants and anxiolytic drugs. Direction of change in basal OCR, reserve capacity, basal ECAR, ATP production and proton leak, where: ↑ = increase compared to vehicle control, ↓ = decrease compared to vehicle control, NR = no response compared to vehicle control. N/A = not available.

Assays		Buspirone	Lorazepam	Citalopram	Fluoxetine	Tianeptine
Acute HepG2 Extracellular Flux Assay	OCR (AC_50_ μM)	>100↓	>100↓	NR	>100↓	>100↓
	Reserve capacity (AC_50_ μM)	43.7↓	NR	NR	70.4↓	21.6↓
	ECAR (AC_50_ μM)	NR	NR	NR	>100↑	NR
	ATP production (AC_50_ μM)	>100↓	>100↓	NR	>100↓	>100↓
	Proton leak (AC_50_ μM)	NR	15↑	>10↓	NR	NR
	Summary mechanism	Substrate inhibitor	Other	Other	ETC inhibitor	Substrate inhibitor
Permeabilized HepG2 Extracellular Flux Assay (OCR)	Most sensitive mechanism (AC_50_ μM)	pyruvate respiration↓ >100	N/A	N/A	pyruvate respiration↓ >100	NR
Glu/Gal assay (cell viability reduction, 24 h incubation treatment)	Glucose (AC_50_ μM)	NR	NR	>10↓	13.3↓	NR
	Galactose (AC_50_ μM)	99↓	NR	>10↓	17.5↓	57.6↓
	Fold Change	UD	NR	UD	0.76	UD
C_max_ (μM)		0.003	0.1	0.1	0.04	0.62

## Data Availability

Data will be made available upon request.
